# The *Ubx* Polycomb response element bypasses an unpaired *Fab-8* insulator via *cis* transvection in *Drosophila*

**DOI:** 10.1371/journal.pone.0199353

**Published:** 2018-06-21

**Authors:** Danfeng Lu, Zhuoran Li, Lingling Li, Liping Yang, Guijun Chen, Deying Yang, Yue Zhang, Vikrant Singh, Sheryl Smith, Yu Xiao, Erlin Wang, Yunshuang Ye, Wei Zhang, Lei Zhou, Yikang Rong, Jumin Zhou

**Affiliations:** 1 Key Laboratory of bioactive peptides of Yunnan Province/ Key Laboratory of Animal Models and Human Disease Mechanisms of Chinese Academy of Sciences & Yunnan Province, Kunming Institute of Zoology, Chinese Academy of Sciences, Kunming, China; 2 Kunming College of Life Science, University of Chinese Academy of Sciences, Kunming, China; 3 Graduate School, University of Chinese Academy of Sciences, Beijing, China; 4 State Key Laboratory of Bio-control, Institute of Entomology, School of Life Sciences, Sun Yat-sen University, Guangzhou, China; 5 Gene Expression and Regulation Program, The Wistar Institute, Philadelphia, PA, United States of America; 6 Department of Molecular Genetics & Microbiology, University of Florida, Gainesville, FL, United States of America; Centre for Stem Cell Research, UNITED KINGDOM

## Abstract

Chromatin insulators or boundary elements protect genes from regulatory activities from neighboring genes or chromatin domains. In the *Drosophila Abdominal-B* (*Abd-B*) locus, the deletion of such elements, such as *Frontabdominal-7* (*Fab-7*) or *Fab-8* led to dominant gain of function phenotypes, presumably due to the loss of chromatin barriers. Homologous chromosomes are paired in *Drosophila*, creating a number of pairing dependent phenomena including transvection, and whether transvection may affect the function of Polycomb response elements (PREs) and thus contribute to the phenotypes are not known. Here, we studied the chromatin barrier activity of *Fab-8* and how it is affected by the zygosity of the transgene, and found that *Fab-8* is able to block the silencing effect of the *Ubx* PRE on the DsRed reporter gene in a CTCF binding sites dependent manner. However, the blocking also depends on the zygosity of the transgene in that the barrier activity is present when the transgene is homozygous, but absent when the transgene is heterozygous. To analyze this effect, we performed chromatin immunoprecipitation and quantitative PCR (ChIP-qPCR) experiments on homozygous transgenic embryos, and found that H3K27me3 and H3K9me3 marks are restricted by *Fab-8*, but they spread beyond *Fab-8* into the DsRed gene when the two CTCF binding sites within *Fab-8* were mutated. Consistent with this, the mutation reduced H3K4me3 and RNA Pol II binding to the DsRed gene, and consequently, DsRed expression. Importantly, in heterozygous embryos, *Fab-8* is unable to prevent the spread of H3K27me3 and H3K9me3 marks from crossing *Fab-8* into DsRed, suggesting an insulator bypass. These results suggest that in the *Abd-B* locus, deletion of the insulator in one copy of the chromosome could lead to the loss of insulator activity on the homologous chromosome, and in other loci where chromosomal deletion created hemizygous regions of the genome, the chromatin barrier could be compromised. This study highlights a role of homologous chromosome pairing in the regulation of gene expression in the *Drosophila* genome.

## Introduction

Eukaryotic genomes are organized by CCCTC-binding factor (CTCF) and other architectural proteins into various functional units and topological domains, where genes and their regulatory information usually reside within these domains [1–3]. The interactions among *cis*-regualtory elements and their binding proteins translate into spatial and temporal specificity of gene expression [4–7]. The *Drosophila* Bithorax complex (BX-C) contains three Hox genes *Ultrabithorax* (*Ubx*), *abdominal-A* (*abd-A*) and *Abdominal-B* (*Abd-B*), and they together determine body segment identity along the anterior-posterior axis. These genes are regulated by parasegment-specific regulatory domains, which are composed of enhancers, and silencers, or Polycomb response elements (PREs), and other types of *cis* elements [8]. These domains are separated by chromatin boundaries or insulators, such as *Frontabdominal* (*Fab*)*-7* and *Fab-8* in the *Abd-B* locus [9–15]. When tested in transgenic flies, these boundary elements could block the effect of a PRE or an enhancer on an insulated promoter [4, 5, 14, 15]. However, in their endogenous locations, these elements do not appear to interfere with enhancer-promoter interactions, possibly due to the existence of “anti insulator” elements near the *Fab-7* and *Fab-8* [16, 17], and only act as chromatin barriers to prevent the regulatory activities on one side from affecting those of a neighboring domain.

Globally, chromatin insulators function as architectural elements to organize the chromosome into a series of topological independent looped domains through direct physical contacts between the insulators [5, 8, 18]. The enhancer-blocking and barrier activities of several insulators are separable into different DNA sequences, for example, the chicken *β-globin* insulator and the *Drosophila* SF1 boundary [19], but these two activities are inseparable for other insulators such as *Fab-8*, which interacts with CTCF, Centrosomal Protein 190 (CP190) and ENY2 proteins [4, 6, 20–23]. CTCF could demarcate the boundaries of H3K27me3 marked repressed chromatin initiated by the Polycomb Group (PcG) proteins. In the *Abd-B* locus, CTCF also interacts with other boundaries including *Miscadastral* (*Mcp*) and *Fab-6* [7, 21, 23].

Intragenic complementation between chromosomes exerts both positive and negative effects on gene function by *trans*-regulatory interactions [24], which are best illustrated by a phenomenon called transvection first described by EB Lewis in 1954 for genetic complementation between *bx*^*34e*^ and *Ubx*^*1*^, two mutant alleles of *Ubx* gene. Here, an enhancer from one allele could act in *trans* on the promoter of a paired second allele [25]. In contrast, a reporter gene could be silenced in *trans* by a PRE inserted at the same site on the homologous chromosome [26]. Thus, transvection requires the close physical proximity of the two homologous alleles.

In *Abd-B*, deletions of *Fab-7* or *Fab-8* resulted in dominant gain of function (GOF) phenotypes, presumably due to the loss of chromatin barriers on the mutant chromosome resulting in the misregulated allele [5, 9,13, 18]. However, whether transvection may contribute to the GOF phenotypes is not known. To address this question and to determine whether a PRE could bypass an insulator in *cis*, we constructed transgenes to analyze the effectiveness of the *Fab-8* insulator in blocking the spread of silenced chromatin marks from *Ubx* PRE in both homozygous (two copies of) and heterozygous (one copy of) transgenic flies. We found that in the homozygous state, the transgenic *Fab-8* blocks the silencing effect of *Ubx* PRE as the DsRed gene is strongly expressed, while in the transgene where CTCF binding sites were mutated in *Fab-8*, the blocking effect is mostly absent and the DsRed gene is minimally expressed. At molecular level, when the transgene is present on both chromosomes, *i*.*e*., when the transgene is homozygous, the spread of H3K27me3 and H3K9me3 are prevented, and H3K4me3 and RNA Pol II are enriched at DsRed gene promoter.

In contrast, when the transgene is heterozygous (mimicking a hemizygous situation in the endogenous locus), *i*.*e*., when the transgene is present only on one copy of the chromosome, the blocking effect is absent. The DsRed expression is at a much lower level, similar to that of the transgene carrying *Fab-8* with CTCF binding sites mutations. The silenced chromatin marks are present across the entire transgene, and the active H3K4me3 mark is largely absent in the promoter and coding region of the DsRed reporter gene. These results suggest that the PRE could bypass the intervening *Fab-8* chromatin barrier through “looped out” type of *cis* transvection, and suggest that, in the endogenous *Abd-B* locus, the deletion of the *Fab* boundary in one chromosome could lead to the similar type of insulator bypass in the wild-type chromosome. Thus this “looped out” type of *cis* transvection could contribute to the GOF phenotypes, and the regulation of the chromatin structure in *Abd-B*.

## Results

### The PRE-mediated repression could bypass the barrier activity of the *Fab-8* insulator when the transgene is heterozygous

**Fig 1:**
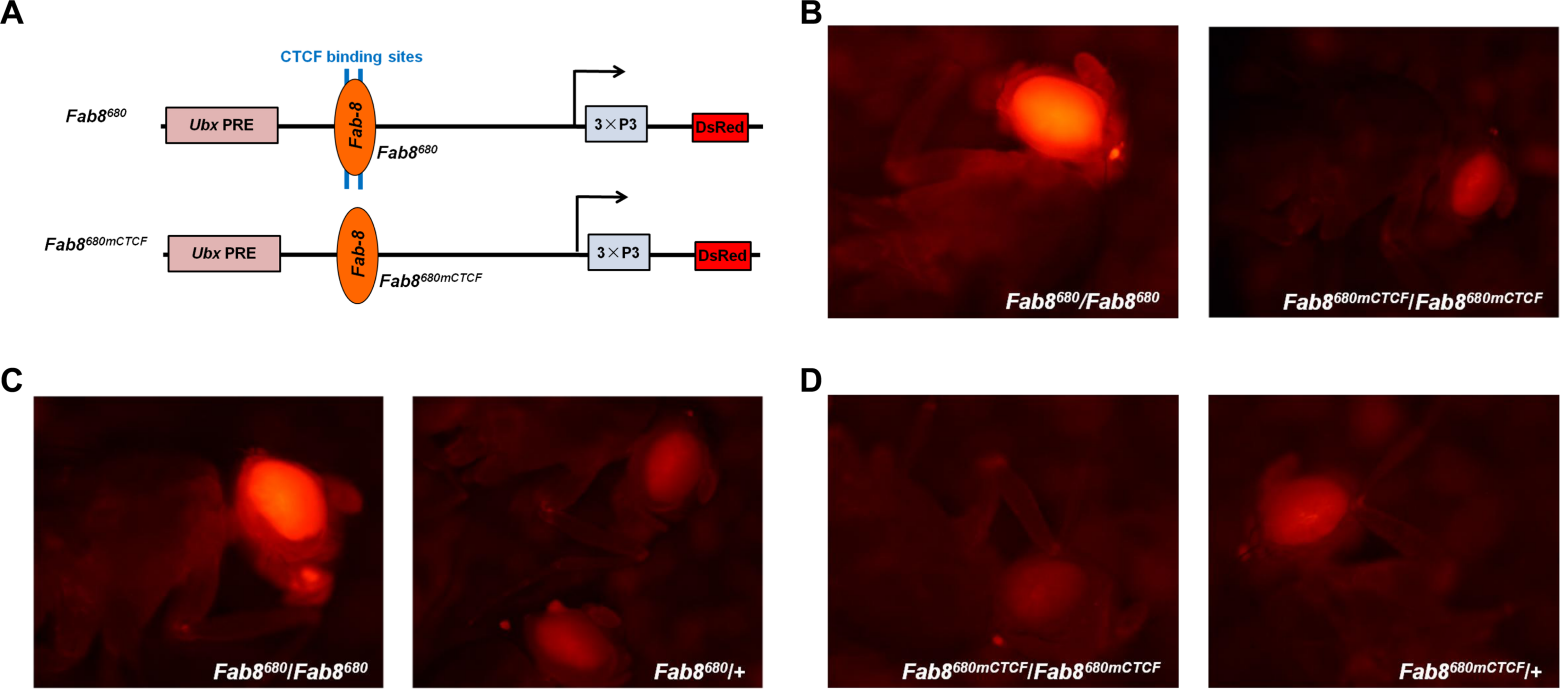
The phenotypes of heterozygous (one copy of) or homozygous (two copies of) *Fab8*^*680*^ transgenic adult flies or *Fab8*^*680mCTCF*^ transgenic adult flies lacking the two CTCF binding sites. **(A)** Schemes of the *Fab8*^*680*^ construct and the *Fab8*^*680mCTCF*^ construct lacking the two CTCF binding sites. Eye-specific 3×P3-DsRed was used as the reporter gene. The PRE from the *Ubx* regulatory region was placed upstream of 3×P3-DsRed to initiate the formation of facultative heterochromatin. The 680 bp tested fragment was cloned between the reporter gene and the PRE and was flanked by two FRT sequences. The transgenic flies were generated by site-specific integration and crossing with the wild-type *y*^*1*^*w*^*1118*^ flies. **(B)** The *Fab8*^*680mCTCF*^ does not block the spread of *Ubx* PRE-mediated repression in homozygous transgenic adult flies. **(C)** Heterozygous *Fab8*^*680*^ adult flies (*Fab8*^*680*^/+) show a loss of the PRE blocking activity from a single *Fab-8* insulator compared with homozygous *Fab8*^*680*^ adult flies (*Fab8*^*680*^/*Fab8*^*680*^). **(D)** The eye colors of homozygous *Fab8*^*680mCTCF*^ transgenic adult flies (*Fab8*^*680mCTCF*^/*Fab8*^*680mCTCF*^) are lighter than their siblings that have one copy of the transgene (*Fab8*^*680mCTCF*^/+). The levels of DsRed expression were analyzed in heterozygous and homozygous adult flies through Nikon AZ100 zoom microscope [3].

To study the effectiveness of the *Fab-8* insulator in blocking the spread of silenced chromatin marks, we took the 661 bp PRE from the regulatory region of *Ultarbithorax* (*Ubx* PRE) [21, 27] and placed it upstream of 3×P3-DsRed [28]. Between the two a 680 bp fragment of the *Fab-8* insulator (*Fab8*^*680*^; Fig 1A) was inserted. Transgenic flies were obtained by *attP* site-specific integration to allow direct comparison of the PRE properties within the same chromatin environment [29]. To verify that CTCF protein is responsible for the PRE blocking effect [21], we used a CTCF binding sites mutant *Fab8*^*680mCTCF*^ (Fig 1A) described previously [22]. The chromatin barrier activity of *Fab8*^*680*^ permitted high level expression of DsRed, resulting in intense level of DsRed signal in the adult eyes, but the *Fab8*^*680mCTCF*^ mutation resulted in a significant reduction of DsRed signal in the eyes of homozygous transgenic flies (Fig 1B). This result suggests that CTCF binding to *Fab-8* interfered with the spread of *Ubx* PRE-mediated silencing, confirming a previous observation [21].

When we compared the DsRed expression between adult flies homozygous (two copies of the transgene) and heterozygous (one copy of the transgene) for the *Fab8*^*680*^ transgene, we noted that the DsRed expression is extremely low in the heterozygous adult flies (Fig 1C). In these flies, the level of DsRed is similar to that of the transgenic *Fab8*^*680mCTCF*^ flies (comparing Fig 1C with Fig 1D), suggesting that DsRed is inhibited when the transgene is in the heterozygous state. We reasoned that the inhibition is due to the *Ubx* PRE bypassing the interposed *Fab-8* insulator through *cis* transvection, whereby the pairing of homologous chromosomes leads to a “looped out” conformation of the transgene when it is in the heterozygous state, as the transgene has no homologous sequences to pair with. As a result, this could create a physical proximity between transgenic sequences located on the opposite sides of the insulator. However, the insulator bypass we observed here is different from what has been reported [30–32]. Chromosome pairing is known to increase the effect of Polycomb-mediated gene silencing, since transgenes containing a PRE often show increased repression when the insertion is present in two allelic copies [25]. Indeed, homozygous *Fab8*^*680mCTCF*^*/Fab8*^*680mCTCF*^ adult flies had noticeably lower DsRed expression than their siblings that have only one copy of the *Fab8*^*680mCTCF*^ transgene (Fig 1D).

**Fig 2:**
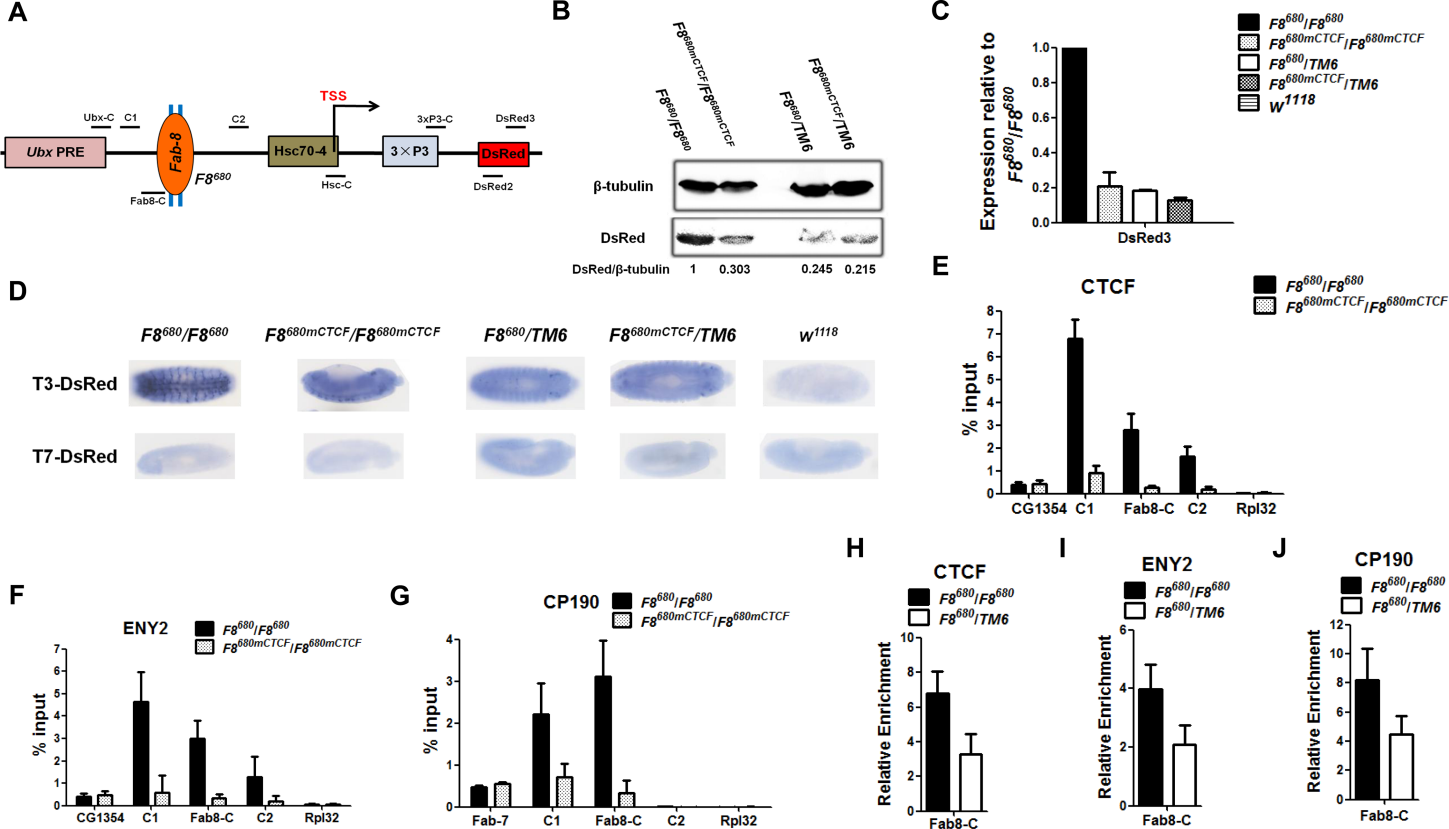
When the transgene is on both chromosomes, the barrier activity of CTCF-dependent *Fab-8* insulator is much stronger than when one copy of the transgene is present. **(A)** Reductive scheme of transgenic *F8*^*680*^ construct in this study and the symbols such as Ubx-C indicate the regions amplified by ChIP-qPCR. Two blue rectangles represent the two CTCF binding sites of the *F8*^*680*^ transgene. TSS represents transcription start site. **(B)** Western blot was performed with 20–24 h transgenic embryos. The protein expression levels of DsRed were significantly decreased in heterozygous transgenic embryos and homozygous *F8*^*680mCTCF*^/*F8*^*680mCTCF*^ transgenic embryos lacking both CTCF binding sites. **(C)** RT-qPCR was done with 20–24 h transgenic embryos. The relative mRNA levels of the 3×P3-DsRed reporter gene were significantly reduced in heterozygous transgenic embryos and homozygous *F8*^*680mCTCF*^/*F8*^*680mCTCF*^ transgenic embryos. The data are expressed as the Mean ± SD using three biological replicates. RNA level was normalized to the housekeeping gene Tubulin. **(D)** 2–14 h transgenic embryos are stained by *in situ* hybridization with antisense probes recognizing DsRed mRNA. T7-DsRed probe and *w*^*1118*^ embryos were used as negative controls. CTCF **(E)**, ENY2 **(F)**, or CP190 **(G)** protein binding to the constructs from homozygous transgenic embryos was analyzed by ChIP followed by real-time quantitative PCR (ChIP-qPCR). Each ChIP-qPCR expreiment with chromatin isolated from 20–24 h transgenic embryos was performed in at least three independent replicates. CG1354 region is the endogenous positive binding region for CTCF and ENY2 proteins. *Fab-7* region was used as a positive control for CP190 binding region and the Rpl32 coding region was used as a negative control. **(H)** CTCF binding to the constructs from homozygous *F8*^*680*^/*F8*^*680*^ or heterozygous *F8*^*680*^/*TM6* transgenic embryos was analyzed by ChIP-qPCR. Relative enrichment is presented as a percentage of input DNA normalized relative to the positive control CG1354 region. ENY2 **(I)** or CP190 **(J)** binding to the constructs from homozygous *F8*^*680*^/*F8*^*680*^ or heterozygous *F8*^*680*^/*TM6* transgenic embryos was analyzed by ChIP-qPCR. Relative enrichment is presented as a percentage of input DNA normalized relative to the positive control CG1354 region **(I)** or *Fab-*7 region **(J)**. The data are expressed as the Mean ± SD. % input is expressed as background immunoprecipitation being subtracted from normalized specific antibody ChIP signals at each position.

To characterize the insulator bypass effect, we generated transgenic embryos, where the DsRed gene is driven by a ubiquitous Hsc70-4 promoter (Fig 2A and S5 Fig) such that the chromatin structure could be analyzed. After integration into the *86F(III)* landing site on the third chromosome [33], we obtained heterozygous *F8*^*680mCTCF*^*/TM6* flies carrying the *F8*^*680mCTCF*^ transgene lacking the two CTCF binding sites and *F8*^*680*^*/TM6* flies carrying the *F8*^*680*^ transgene with the intact CTCF binding sites. Then the heterozygous offspring were mated to obtain homozygous *F8*^*680mCTCF*^*/F8*^*680mCTCF*^ and *F8*^*680*^*/F8*^*680*^ transgenic flies. Although the expression of the DsRed gene in these embryos from the transgenic flies was not high enough to allow consistent detection of DsRed under fluorescent microscope, the results of RT-qPCR and Western blot from the transgenic embryos indicated that the DsRed gene is expressed. Our results further indicated that heterozygous embryos for the *F8*^*680*^ construct showed much lower level expression of DsRed compared with homozygous embryos for the *F8*^*680*^ transgene, which is consistent with the observation made with adult flies (Fig 2B and 2C, S2A–S2C Fig). *In situ* hybridization with T3- or T7-labeled antisense probes recognizing DsRed mRNA showed that DsRed mRNA is expressed at a higher level in homozygous *F8*^*680*^*/F8*^*680*^ transgenic embryos than in heterozygous transgenic embryos (Fig 2D and S2D Fig). These results demonstrated that when the transgene insertion is on both chromosomes, the barrier activity is much stronger than when a single copy of the transgene is present, again suggesting that the silencing activity of the PRE could bypass the *Fab-8* insulator.

As *Fab-8* also interacts with CP190, ENY2 and several other proteins [20, 21, 23, 25], we did Chromatin immunoprecipitation (ChIP) analysis and confirmed that CTCF binds to the *F8*^*680*^ fragment in homozygous animals carrying the *F8*^*680*^ transgene, while CTCF protein was hardly detected in *F8*^*680mCTCF*^*/F8*^*680mCTCF*^ homozygous animals (Fig 2E). Similarly, both CP190 and ENY2 proteins interact with the transgenic *F8*^*680*^ insulator, and the binding of these two proteins were reduced if CTCF binding sites are mutated (Fig 2F and 2G), confirming that CTCF association is required to recruit CP190 or ENY2 protein [20, 21]. Importantly, in heterozygous *F8*^*680*^*/TM6* transgenic embryos, CTCF still interacts with *Fab-8* although there is a decreased binding to the transgenic *F8*^*680*^ insulator due to the presence of one copy of the *F8*^*680*^ (Fig 2H), suggesting that the insulator bypass is not due to the inactivation of *Fab-8*. Similarly, both CP190 and ENY2 proteins are also recruited to the transgenic *F8*^*680*^ insulator in heterozygous *F8*^*680*^*/TM6* embryos (Fig 2I and 2J).

### *Fab-8* restricts the spread of repressive chromatin marked by trimethylated H3K27 and H3K9 in homozygous transgenic embryos

**Fig 3:**
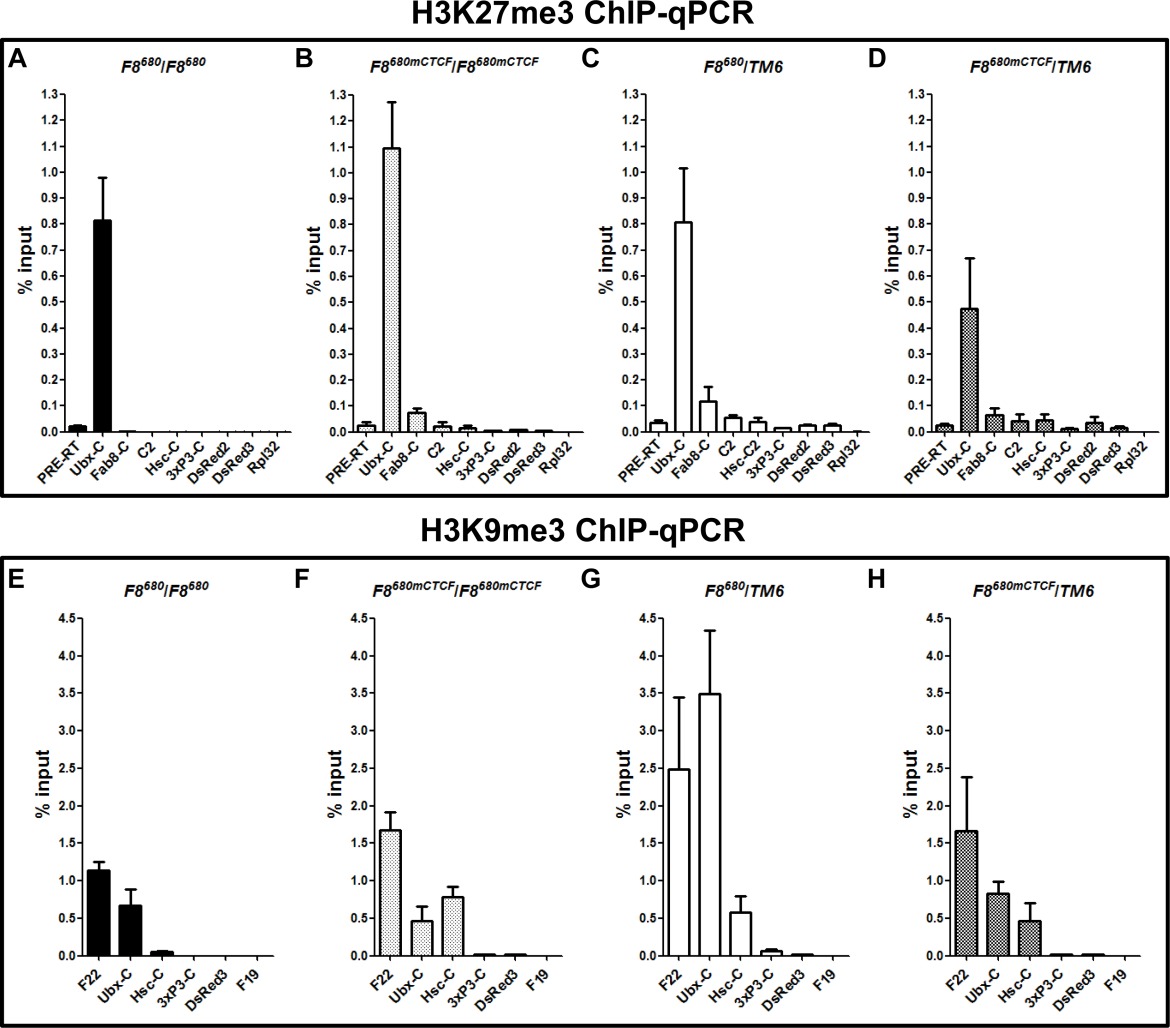
*Fab-8* blocks the propagation of *Ubx* PRE-mediated repressive H3K27me3 and H3K9me3 marks in homozygous transgenic embryos. **(A, B, C, D)** The ChIP-qPCR expreiments were performed with chromatin isolated from 20–24 h transgenic embryos. PRE-RT region or Rpl32 coding region was used as a positive control or a negative control for H3K27me3 binding. **(E, F, G, H)** H3K9me3 ChIP-qPCR expreiments were performed with chromatin isolated from 20–24 h transgenic embryos. The heterochromatic control region F22 and the intergenic region of euchromatin F19 were used as a positive control and a negative control for H3K9me3 ChIP-qPCR, respectively. Background immunoprecipitation was subtracted from normalized specific antibody ChIP signals at each position examined.

To characterize the PRE blocking effect of *Fab-8*, we examined the changes in chromatin structure of the transgenic constructs in heterozygous and homozygous *F8*^*680*^ and *F8*^*680mCTCF*^ transgenic embryos. We first used ChIP-qPCR in the transgenic embryos to examine the association of the *Ubx* PRE-mediated H3K27me3 mark with different regions of the transgene (Fig 2A). In homozygous transgenic embryos containing the *F8*^*680*^, H3K27 trimethylation initiated by the *Ubx* PRE is restricted to the vicinity of the PRE region, suggesting the barrier function is intact (Fig 3A). But in *F8*^*680mCTCF*^ transgenic embryos, the H3K27me3 mark is distributed in the entire transgene (Fig 3B and 3D). Importantly, in heterozygous embryos carrying the *F8*^*680*^ transgene, the H3K27me3 mark also spreads across the entire transgene, indicating that the *Fab-8* insulator fails to block the silencing effect of the *Ubx* PRE in this setting (Fig 3C).

We also tested for the presence of H3K9me3 on the transgene and found that its binding profile is highly similar to that of H3K27me3: In the chromatin from homozygous *F8*^*680*^*/F8*^*680*^ transgenic embryos, H3K9me3 signals are high in the vicinity of the PRE but they are reduced to low level in the promoters and gene body region of the reporter gene DsRed (Fig 3E). In contrast, in homozygous *F8*^*680mCTCF*^*/F8*^*680mCTCF*^ transgenic embryos, H3K9me3 is present at high level across the entire transgene (Fig 3F). Similar to the H3K27me3 mark, in heterozygous transgenic embryos, the H3K9me3 mark also extends through the entire transgene (Fig 3G and 3H). These results suggest that the spread of H3K27me3 and H3K9me3 is limited by the barrier activity of the *Fab-8* insulator, but this restriction occurs only when the transgene is in the homozygous condition. In the heterozygous setting, the barrier activity is lost and these chromatin marks are indistinguishable from those of the transgene carrying the *F8*^*680mCTCF*^.

### The H3K27me3 mark covers the entire transgene in heterozygous embryos in spite of *Fab-8*

**Fig 4:**
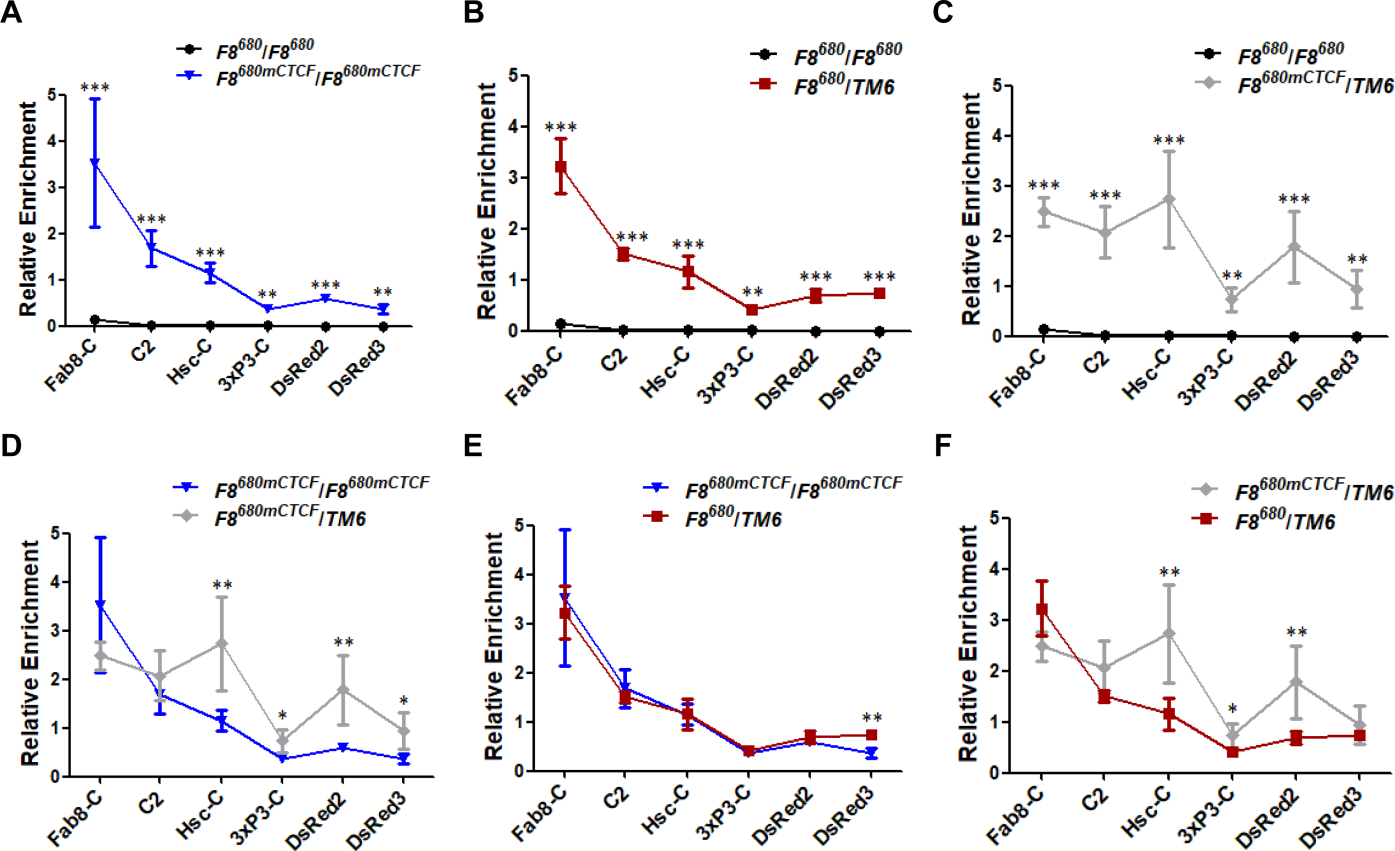
H3K27me3 mark is propagated through bypassing the interposed *Fab-8* located in *cis* to the promoter regions of the transgene in heterozygous embryos. According to the results of H3K27me3 ChIP-qPCR from **Fig 3A–3D**, relative enrichment is presented as a percentage of input DNA normalized relative to the endogenous region PRE-RT from the *Ubx* gene. Relative enrichment of the H3K27me3 mark is higher in homozygous *F8*^*680mCTCF*^/*F8*^*680mCTCF*^ transgenic embryos **(A)**, heterozygous *F8*^*680*^/*TM6* transgenic embryos **(B)** or *F8*^*680mCTCF*^/*TM6* heterozygotes **(C)** versus (VS.) homozygous *F8*^*680*^/*F8*^*680*^ transgenic embryos. Homozygous *F8*^*680mCTCF*^/*F8*^*680mCTCF*^ transgenic embryos VS. *F8*^*680mCTCF*^/*TM6* heterozygotes **(D)** and *F8*^*680*^/*TM6* heterozygotes **(E)**, respectively. **(F)** Heterozygous *F8*^*680mCTCF*^/*TM6* transgenic embryos VS. heterozygous *F8*^*680*^/*TM6* transgenic embryos. A 5% or lower *P* value is considered to be statistically significant using Student’s *t*-test. The data are expressed as the Mean ± SD. *, *P* < 0.05; **, *P* < 0.01; ***, *P* < 0.001.

Experiment in Fig 1C indicated that the *Fab8*^*680*^ fails to sequester the PRE-mediated gene repression in heterozygous transgenic flies, suggesting that the *Ubx* PRE could bypass the intervening *Fab-8* insulator when the transgene is present on one of the two homologous chromosomes. To further define this insulator bypass phenomenon at molecular level and to deduce proposed working models for this insulator bypass, we compared relative enrichment of the H3K27me3 mark (relative to the endogenous region PRE-RT from the *Ubx* gene) in various regions of the transgene in homozygous *F8*^*680*^*/F8*^*680*^ or *F8*^*680mCTCF*^*/F8*^*680mCTCF*^ transgenic embryos and in heterozygous *F8*^*680*^*/TM6* or *F8*^*680mCTCF*^*/TM6* transgenic embryos (Fig 3A–3D). In homozygous *F8*^*680*^*/F8*^*680*^ transgenic embryos, relative enrichment of the H3K27me3 mark is much lower in the entire transgenic region than that in the embryos carrying the *F8*^*680mCTCF*^ transgene (Fig 4A and 4C). However, relative enrichment of the H3K27me3 mark is higher in heterozygous *F8*^*680*^/*TM6* transgenic embryos than that in homozygous *F8*^*680*^*/F8*^*680*^ transgenic embryos (Fig 4B). These results suggest that either the absence of the interaction between CTCF-dependent *Fab-8* insulators on homologous chromosomes or a simple looping out of the transgene resulted in the spread of Polycomb repression to the entire transgenic region.

Together, Fig 4B, 4C and 4D implied that the *Ubx* PRE-mediated H3K27me3 mark could bypass *Fab-8* placed in *cis* to propagate Polycomb repression to the coding region of the reporter gene in heterozygous transgenic embryos, probably due to the intervening *Fab-8* transgene being “looped out”. The comparison between homozygous *F8*^*680mCTCF*^ transgenic embryos and heterozygous embryos (Fig 4D and 4E) indicated that Polycomb repression could spread directly along the chromatin fiber in homozygous *F8*^*680mCTCF*^*/F8*^*680mCTCF*^ transgenic embryos. Experiments shown in both Fig 4E and 4F provided evidence that Polycomb repression spreads not only along chromatin fiber but also through bypassing the interposed *F8*^*680mCTCF*^ in heterozygous *F8*^*680mCTCF*^/*TM6* embryos.

### *Fab-8* permits the formation of active chromatin and RNA polymerase II elongation on the reporter gene when the transgene is homozygous

**Fig 5:**
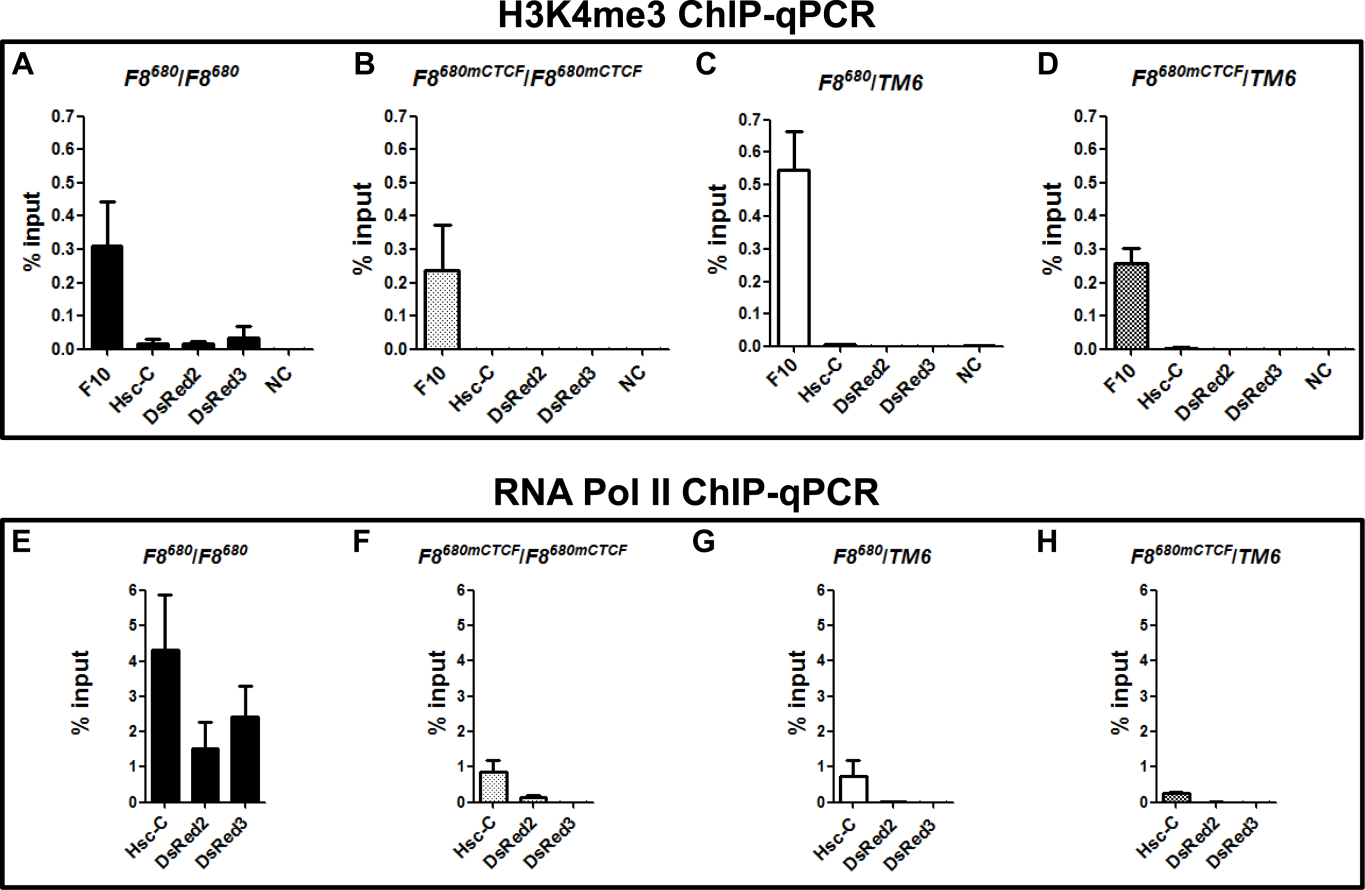
*Fab-8* prevents the *Ubx* PRE-mediated transcriptional silencing of the transgene in homozygous embryos. **(A, B, C, D)** H3K4me3 mark is mostly present in homozygous embryos carrying the *F8*^680^ transgene. F10 region, 1 kb downstream of the active *Ubx* gene, and NC from a gene desert on *Drosophila melanogaster* chromosome 2R were used as a positive control and a negative control for H3K4me3 ChIP-qPCR, respectively. **(E, F, G, H)** RNA Pol II is paused in heterozygous transgenic embryos. The ChIP-qPCR expreiments were performed with chromatin isolated from 20–24 h transgenic embryos. Background immunoprecipitation was subtracted from normalized specific antibody ChIP signals at each position.

To confirm that the *Fab-8* insulator permitted the formation of active chromatin on the DsRed gene, we performed ChIP-qPCR with antibodies directed against H3K4me3 and against RNA polymerase II (Pol II) (S3 Fig), and found that H3K4me3 is localized at the transcription start site (TSS) and gene body of the DsRed gene in homozygous *F8*^*680*^*/F8*^*680*^ transgenic embryos (Fig 5A), but this is not seen in heterozygous *F8*^*680*^/*TM6* transgenic embryos (Fig 5C), nor is it seen in any of the transgenic embryos carrying the *F8*^*680mCTCF*^ (Fig 5B and 5D). Thus, H3K4me3 signals correlate with the transcriptional activity of the Hsc70-4 promoter and the absence of H3K27me3.

Next, we examined the occupancy of RNA Pol II, and found that in *F8*^*680*^*/F8*^*680*^ transgenic embryos, more RNA Pol II binds to the gene body (Fig 5E), indicating more productive elongation. In heterozygous transgenic embryos, RNA Pol II signals near the Hsc70-4 promoter are much higher than those located within the reporter gene (Fig 5G and 5H), suggesting that these signals represent promoter-proximal paused Pol II [34, 35]. In addition, the comparison of RNA Pol II levels enriched by the Hsc70-4 promoter (Fig 5E–5H) indicated that the H3K27me3 marked chromatin has decreased RNA Pol II recruitment to the promoter regions, and reduced the level of the transcription [36]. The fact that more H3K4me3 and RNA Pol II deposited at the Hsc70-4 promoter in homozygous *F8*^*680*^*/F8*^*680*^ transgenic embryos (Fig 5) demonstrated that the promoter regions associated with H3K4me3 mark and RNA Pol II are present in the facultative heterochromatin borders, thus the *Fab-8* insulator has prevented the silencing of the DsRed gene by the *Ubx* PRE.

## Discussion

Currently, how a PRE and an insulator interact in the hemizygous state due to a chromosomal deletion on the homologous chromosomes is not understood. Such a situation is seen in the regulatory mutations, such as *Mcp*, *Fab-7* and *Fab-8* deletion mutants in the *Abd-B* locus, where the wild-type copy of the boundary exists in hemizygous [9, 13]. In this study, we constructed a similar situation in the transgenic flies, where a transgenic PRE, an insulator and a reporter gene are placed in the heterozygous situation. We found that the *Fab-8* barrier activity could be bypassed by the *Ubx* PRE, leading to the silencing of the reporter gene DsRed. However, when the transgene is homozygous, the insulator completely blocks the PRE. This observation is confirmed by analyses of chromatin marks, RNA Pol II binding and gene expression. Our results suggest that in *Abd-B*, insulator bypass by PREs in *cis* could occur and contribute to the phenotypes of *Fab-7* or *Fab-8* mutations.

**Fig 6:**
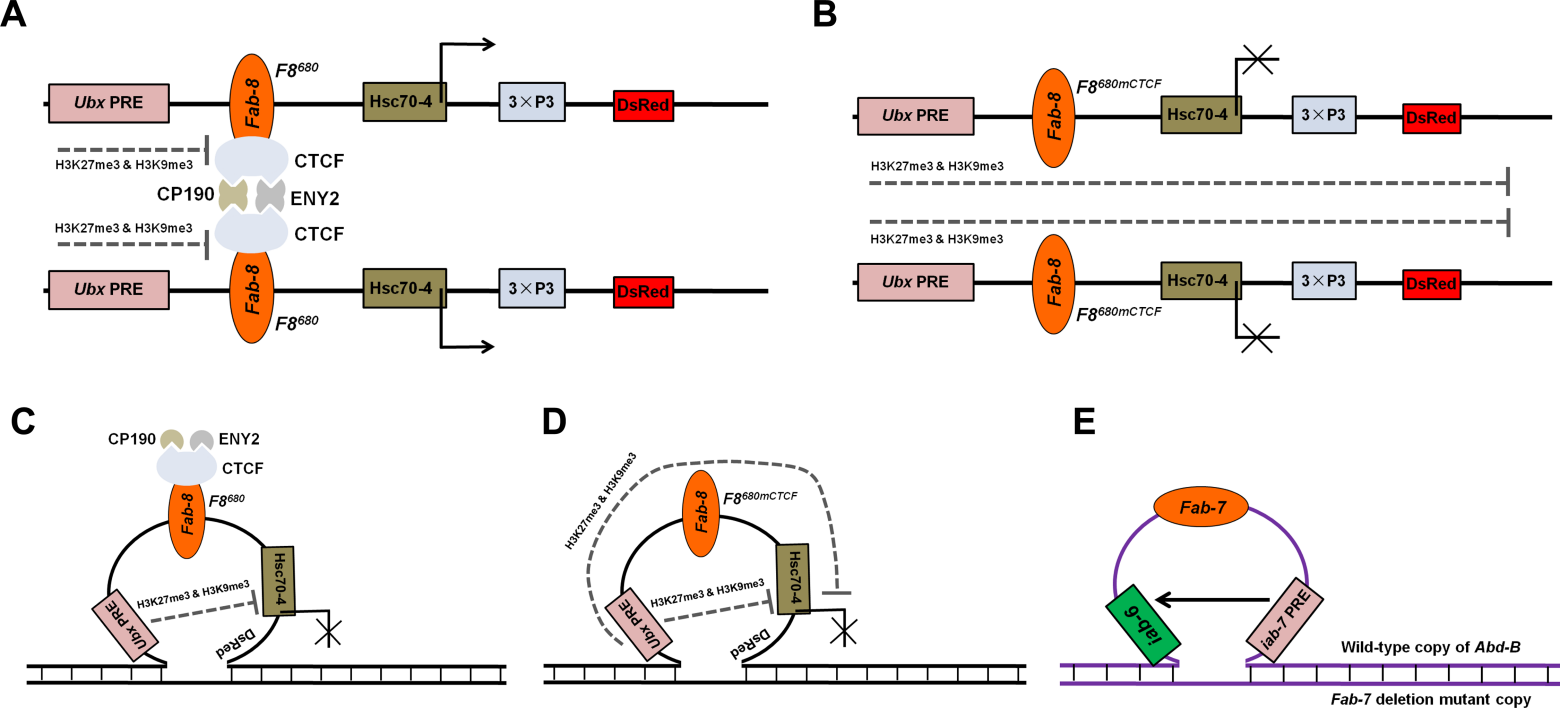
Proposed working models for *Ubx* PRE bypassing chromatin barrier activity of the intervening *Fab-8* insulator located in *cis* in *Drosophila* embryos. **(A)** In the presence of the interactions among the *F8*^*680*^, insulator binding protein CTCF and cofactors such as CP190 and ENY2, the spread of repressive histone modifications from *Ubx* PRE into the neighbouring region is inhibited and thus *Ubx* PRE-mediated repressive chromatin modifications are largely absent in the promoter and coding region while H3K4me3 and RNA Pol II are present instead. **(B)** In homozygous embryos carrying the *F8*^*680mCTCF*^ transgene lacking the two CTCF binding sites, owing to the absence of the interaction between CTCF-dependent *Fab-8* insulators, H3K27me3 and H3K9me3 could spread over nucleosomes, which leads to the formation of inactive chromatin domains and repression of the adjacent DsRed reporter gene. **(C)** In heterozygous embryos carrying the *F8*^*680*^ transgene with the intact CTCF binding sites, independent *F8*^*680*^ transgene has the PRE blocking activity through recruiting CTCF, CP190 and ENY2 proteins to chromatin, but the transgene is unpaired and “looped out” because of the homologous chromosome that is not integrated by the transgenic construct. This homologue pairing-mediated topology allows for bypass of the *F8*^*680*^ chromatin insulator by the *Ubx* PRE to inhibit the transcription of the reporter gene located in *cis*. **(D)** In heterozygous embryos carrying the *F8*^*680mCTCF*^ transgene, the propagation of Polycomb repression along the chromatin fiber and the presence of *Ubx* PRE bypassing the *F8*^*680mCTCF*^ transgene in *cis* could lead to the downregulation of the reporter gene activity. **(E)** In the endogenous *Abd-B* locus, one copy of the *Fab-7* insulator and *iab-7* PRE is deleted. The wild-type copy of *Fab-7* is looped out due to homologue pairing-mediated topology effect, causing the *iab-7* PRE to bypass in *cis* the intact *Fab-7* chromatin boundary to regulate the chromatin in the adjacent *iab-6* regulatory domain. The dashed lines represent the possible direction of repressive H3K27me3 and H3K9me3 marks propagation.

What could account for the difference between the presence of one copy of the transgene and two copies of the transgene? It is known that paired insulators could interact with each other to function more efficiently in flies [37], and also increase the stability of homolog pairing [38]. One possibility is that when the transgene is homozygous, the two *Fab-8* insulators located on homologous chromosomes could pair through the interactions between insulator binding protein and cofactors to block effectively the spread of the PRE-mediated repression (Fig 6A and 6B). We believe in the second possibility that pairing induced conformation change may “loop out” the intervening *Fab-8* insulator, creating the physical proximity between the *Ubx* PRE and the DsRed promoter and thus insulator bypass and gene silencing (Fig 6C and 6D).

Pairing dependent silencing of PREs has been reported, whereby when transgenes containing a PRE are homozygous, the silencing activity is greatly enhanced [25]. Our data clearly showed that paired *Fab-8* blocked the *Ubx* PRE when PREs are paired and presumably are exhibiting pairing sensitive silencing, but we could not compare which forms of PREs silencing, *i*.*e*., pairing dependent versus independent, is more efficiently blocked by *Fab-8*.

Several cases of insulator bypass have been reported. Enhancers and PREs could bypass the blocking effect of two tandem *gypsy* insulators in *cis* [30–32] while Morris et al. [39] found that transvection could take place through an enhancer bypassing an interposed *gypsy* insulator in *cis* in *y*^*2*^*/y*^*3c3*^ flies due to conformational changes in the gene caused by the topology of paired alleles. In this study, we observed a similar mode of transvection by the *Ubx* PRE in heterozygous transgenic embryos (Fig 6C and 6D).

Although transvection is generally permitted throughout the *Drosophila* genome, both PRE elements and enhancers prefer *cis*-target genes to those located in *trans* [24, 25]. In the BX-C, enhancers initiate the homeotic gene expression at about two hours of embryogenesis, and then the PREs maintain the expression patterns during the remainder of the life cycle when the initial expression state is over at about six hours of embryogenesis [9–15, 40, 41]. Homologous chromosome pairing is widespread in the *Drosophila* genome. Pairing of homologous alleles of the BX-C starts as early as two hours of embryogenesis, which is coincident with the onset of robust zygotic transcription, and the pairing frequencies reach a plateau of about 70% in between 6 and 13 hours of development [42, 43]. Thus a pairing dependent insulator bypass by the PRE is clearly possible. Here, We propose that homologue pairing near the transgenic insertion site leads to an unpaired loop formed by the transgene, making the *cis*-linked promoter accessible to the activity of the *Ubx* PRE in spite of the *Fab-8* insulator. Hence, the PRE-blocking effect of one *Fab-8* insulator may be compromised by constraints forced by the looped structure (Fig 6C and 6D). Our “looped out” type of *cis* transvection model predicts that insulator bypass in unpaired regions requires homologous chromosomal pairing and in early *Drosophila* embryos where pairing is weak or absent, an insulator could still block the effect of a silencer as has been observed by Fujioka et al. [44].

Each homeotic gene is controlled by two or more PRE elements and PREs in each *cis*-regulatory domain could maintain the inactive state of this domain [13]. However, in the hemizygous mutations that remove *Fab* in one chromosome, the PREs bypass the intact chromatin boundary elements in *cis* through “looped out” type of transvection to stabilize the silenced state of the adjacent regulatory domains in the *Abd-B* region (Fig 6E). Mihaly et al. [13] found that the classⅠdeletions that remove both the *Fab-7* insulator and *iab-7* PRE have a complete transformation of A6 into a copy of A7 (GOF phenotypes). But as heterozygotes, the classⅠdeletions have weaker GOF phenotypes (there is still a small part of A6 tergites) than their homozygous counterparts. We believe this “looped out” type of *cis* transvection model (Fig 6E) accounts for these weaker GOF phenotypes of the classⅠdeletions in heterozygotes. Thus, pairing dependent insulator bypass could play an important role in the regulation of chromatin structure in *Drosophila* genome.

## Materials and methods

### *Drosophila* stocks, transgenic constructs, germ line transformation and genetic crosses

All flies were cultured at 25°C on the standard *Drosophila* cornmeal, yeast, sugar and agar medium. The promoter of the Hsc70-4 gene (S5 Fig) was obtained by polymerase chain reaction (PCR) using the following primers: 5’GCTCGCTGAAAAAGGCGAA3’ and 5’CTGGAAAGAATTACAACGGTGTG3’, and after digestion with SphI, the 365 bp fragment which includes the TSS was cloned into BarrierTester1 vector [28] containing the eye-specific 3×P3-DsRed reporter gene. 680 bp DNA fragment representing the *Fab-8* insulator (S5 Fig; *F8*^*680*^) was PCR amplified from genomic DNA with the following primers: 5’CGTCAACGCCAACCAGCAC3’ and 5’CCTGGGTTCATTATTTTAAAAC3’ and cloned into the above plasmid after digestion with SpeI. We mutated the two CTCF binding sites in the *F8*^*680*^ fragment (*F8*^*680mCTCF*^; S1 Fig) as described previously [22]. Then the PCR-amplified fragments from the above constructs using the following primers: 5’CATATGCAACCCAAGATAAAAATATCTTTTTC3’ and 5’CTAAAGGAACAGATGGTGGCGT3’ were ligated into pUAST-attB vector (kindly provided by professor Yikang Rong from Sun Yat-sen University) cleaved by HindIII and XbaI. The resulting plasmids were verified by sequencing. In order to make the expression levels comparable, the resulted transgenic constructs were injected separately into preblastoderm embryos and integrated into the *86F(III)* landing site on the third chromosome (*M[3×P3-RFP*.*attP]ZH-86Fb*) (from the Bloomington *Drosophila* stock center; kindly provided by professor Yikang Rong) using *phi*C31 recombination system [33]. The resultant flies were crossed with *w*^*1118*^ flies to take away the *phi*C31 on the chromosome X, and the transgenic progeny were identified by their eye colors. Because the *86F(III)* flies have a 3×P3-RFP on the third chromosome, the 3×P3-RFP cassette was eliminated from line *M[3×P3-RFP*.*attP]ZH-86Fb* by crossing the transgenic progeny with the Cre (*yw; Cyo*, *P[y+*, *cre]/Sco; +*) recombinase-expressing lines. The Cre recombinase induces 100% excisions in the next generation. Subsequently, heterozygous flies containing one copy of the transgene (named *F8*^*680mCTCF*^/*TM6* for the *F8*^*680mCTCF*^ transgene lacking the two CTCF binding sites, or *F8*^*680*^/*TM6* for the *F8*^*680*^ transgene with the intact CTCF binding sites; S4 Fig) were yielded by crossing with the line *C(1;Y)1*, *Y[1]; Sb[1]/TM6*. Then males and females of the heterozygous offspring were interbred to obtain homozygous flies carrying two copies of the transgene (named *F8*^*680mCTCF*^*/F8*^*680mCTCF*^ or *F8*^*680*^*/F8*^*680*^; S4 Fig).

### Western blot analysis

Dechorionated transgenic embryos (0.5 μL per embryo) or adult flies (10 μL per fly) were crushed with a pestle in RIPA lysis buffer containing SDS loading buffer and boiled for 10 min followed by centrifugation at 13,000 g for 10 min. The supernatants were transferred to clean tubes and samples were loaded into 10% running SDS-PAGE gel, and the proteins were transferred to a PVDF membrane (Millipore) using wet-blot. After being blocked for 1 h at room temperature (RT) with blocking solution containing 5% skimmed milk in PBST (0.1% Tween-20 in PBS, pH7.4), the membrane was incubated with primary antibody in PBST containing 3% skimmed milk overnight at 4°C and then washed 3 times with PBST followed by incubation with 1:5000 HRP-labeled secondary antibody (CST) for 1 h at RT. After the membrane being washed 3 times with PBST, the ECL signal was recorded using Immobilon Western Chemiluminescent HRP Substrate (Millipore) and Tanon-4200SF Chemiluminescent Imaging System (Tanon). The following primary antibodies were used: rabbit anti-RFP (1:4000; ab62341), mouse anti-β-tubulin (1:15,000; Transgen).

### Total RNA extraction and RT-qPCR analysis

RNA isolation of dechorionated transgenic embryos or adult flies was done using Trizol Reagent according to the manufacturer’s instructions (TaKaRa). After chloroform extraction, the RNA was precipitated with isopropanol. The pellet was washed with 75% Ethanol and resuspended in DEPC-water. For reverse transcription, 1 μg of the generated RNA was incubated with reverse transcriptase (PrimeScript RT reagent Kit with gDNA Eraser, TaKaRa). Real-time qPCR was performed using a Roche LightCycler 480 equipment and LightCycler 480 SYBR Green I Master (Roche). Reactions were performed in a total reaction volume of 10 μL using cDNA template and forward and reverse primers. Thermocycler conditions were as follows: 95°C for 5 min and then 40 cycles of 95°C for 15 seconds, 60°C for 15 seconds and 72°C for 15 seconds. Following the amplification process, a melt curve was generated between 60°C and 95°C. Ct values were determined using the advanced relative quantification method and then exported to further analyze the expression levels. Data were analyzed using the 2^-△△Ct^ method. The expression level for each investigated gene was normalized to housekeeping genes (Tubulin or Rpl32) and then to the expression of homozygous *F8*^*680*^*/F8*^*680*^ transgenic flies. Primers sequences are given in S1 Table.

### Chromatin immunoprecipitation (ChIP) assays

20–24 h transgenic embryos were collected by washing with distilled water, dechorionated with 50% bleach for 4 min and then frozen in liquid nitrogen (N_2_) and stored at -80°C before use. Chromatin from frozen transgenic embryos was isolated and immunoprecipitated as described previously with some modifications [2, 21, 27–29, 44]. Briefly, 150–200 mg of the initial material was collected for each experiment. The material was ground in N_2_ using a mortar as N_2_ evaporates. Homogenate in powder form was suspended in ice-cold PBS buffer. Crosslinking was performed for 15 min in the presence of 1.8% formaldehyde after tissue homogenization. The reaction was stopped by adding glycine (final concentration of 200 mM). The homogenate was cleared by passing through 100 μm Nylon filter Cell Strainer (BD Falcon) and pelleted by centrifugation at 4,000 g at 4°C for 8 min. All subsequent steps were performed on ice. Cells were washed twice in ice-cold PBS and resuspended in ChIP lysis buffer (50 mM Tris-HCl, pH8.0, 150 mM NaCl, 5 mM CaCl_2_, 0.5 mM DTT, 1% Triton X-100, 0.1% SDS, 1×EDTA-free Protease Inhibitor Cocktail (Bimake) and 1×Phosphatase Inhibitor Cocktail (Bimake)). Cells were lysed for 20 min on a rotating wheel at 4°C and micrococcal nuclease (MNase, CST) was used to break chromatin into fragments with an average length of 180 bp-900 bp. For MNase fragmentation, the cells in 500 μL ChIP lysis buffer were briefly sonicated in a Diagenode Bioruptor (Denville, NJ) for 10 cycles (high intensity, 30 s on, 30 s off) and then digested for 15 min with 1 μL MNase at 37°C. Digestion was stopped by moving the tubes to ice and adding 20 μL of 500 mM EDTA. The material was pelleted by centrifugation at 12,000 g for 5 min, and the supernatant fluid was transferred to a new tube. The pellet was treated with the second 500 μL of ChIP lysis buffer, and the preparation was centrifuged at 12,000 g for 5 min. The two portions of the supernatant fluid were pooled, cleared by centrifuging at 12,000 g for 10 min, and the resultant chromatin extract (1 mL) was used in four ChIP experiments. One aliquot of chromatin extract was kept as a control sample (input).

ChIP experiments involved incubation with mouse antibodies against CTCF [7], CP190 and ENY2 (GL Bio, Shanghai); mouse anti-RNA Pol II (8WG16, Covance); mouse anti-H3K27me3 (ab6002, Abcam); H3K9me3 (ab8898, Abcam) and H3K4me3 (JBC1888194, 07–473, Millipore). Corresponding non-immune IgG (CST or Abcam) was used as a nonspecific antibody control. Samples containing about 10 μg of DNA equivalent in 1 mL of ChIP lysis buffer were incubated overnight at 4°C with 3 μg antibody on a rotating wheel. Antibody-chromatin complexes were collected using protein G-magnetic beads (Life) at 4°C over 7 h. After three rounds of washing with low salt buffer, once with high salt buffer and TE buffer (10 mM Tris-HCl, pH8.0, 1 mM EDTA), the DNA was eluted with elution buffer (100 mM NaHCO_3_, 1% SDS) and the crosslinks were reversed by incubating overnight at 65°C. And then the samples were phenol-chloroform extracted after treatment with RNase A and Proteinase K and ethanol precipitated in the presence of 110 mM sodium acetate and 5 μL of Acryl Carrier (Solarbio). Immunoprecipitated DNA was dissolved in 50 μL of DEPC-water.

At least three independent chromatin preparations were made for each ChIP sample. For ChIP-qPCR, after immunoprecipitation and DNA purification, enrichment of specific DNA fragments was analyzed by real-time qPCR using the Roche LightCycler 480 equipment and a LightCycler 480 SYBR Green I Master kit. The intensity of ChIP signals (% input) is expressed as background nonspecific IgG immunoprecipitation being subtracted from normalized specific antibody ChIP signals at each position examined [27]. Primers used in ChIP-qPCR analysis are listed in S2 Table.

### Probes generation and RNA *in situ* hybridization

The coding region of DsRed gene was obtained from BarrierTester1 vector by PCR using the following primers: 5’ATGAGGTCTTCCAAGAATGTTATC3’ and 5’CTAAAGGAACAGATGGTGGC3’. After digestion with HindIII and BamHI, the 678 bp fragment was cloned into pBlueScript SK(+) vector and verified by sequencing. To generate T7 probe or T3 probe, pBlueScript SK(+)-DsRed plasmid was digested with BamHI or SalI, and then purified and subjected to *in vitro* transcription and DIG-labeling (Roche DIG RNA Labeling Mix) following the manufacturer’s instructions. After DNase digestion to remove template DNA and ethanol-precipitation, the probes resuspended in DEPC-water were stored at -80°C until ready to use.

Whole-mount RNA *in situ* hybridization was done as described previously [16, 17, 40]. Briefly, staged transgenic embryos were collected, dechorionated with 50% bleach for 4 min, and fixed in formaldehyde-saturated heptane for 20 min and stored at -20°C in methanol until further use. For the *in situ* hybridization, the embryos were treated in xylene to remove lipids and digested with Proteinase K to remove proteins. Subsequently, the embryos were fixed again with 5% formaldehyde for 20 min at RT on a nutator and then washed five times in PBT (1×PBS, 0.1% Tween80). After the last wash the embryos were incubated for 10 min on a nutator in a prehybridization buffer (50% formamide, 5×SSC, 50 μg/mL heparin, 0.1% Tween80). Before adding the probe, the embryos were incubated for 1–2 h at 55°C in hybridization buffer (prehybridization buffer containing 200 μg/mL yeast tRNA). The probe was denatured to prevent secondary structures at 80°C, snap cooled and added at a final concentration of 2 ng/μL in hybridization buffer to the embryos. The embryos were then incubated for more than 14 h at 55°C. The embryos were washed five times with prehybridization buffer for 1 h at 55°C and then six times with PBT for 10 min at RT. Next, the embryos were blocked with 5% BSA in PBT for 1 h on the nutator and anti-DIG-AP antibody (Roche) was added to the embryos at a 1:2000 dilution with 1% BSA in PBT. The antibody was incubated for 2 h or overnight. Afterwards the embryos were washed four times for 15 min in PBT. Color reaction was carried out at RT with AP staining buffer containing NBT and BCIP (Roche). Stained embryos were rinsed in PBT, washed ten times with 100% Ethanol and mounted on glass slides.

### Statistical analysis

Data were expressed as the Mean ± standard deviations (SD) where indicated, and a 5% or lower *P* value is considered to be statistically significant using Student’s *t*-test. All statistical analyses were performed with GraphPad Prism 5.0 software.

## Supporting information

S1 Fig CTCF protein could bind to *F8*^*680*^. Biotin-DNA pulldown assay indicated that the *F8*^*680mCTCF*^ lacking the two CTCF binding sites does not bind to GST-CTCF. Experimental setting for Biotin-DNA pulldown assay is described in S1 Method.
(TIF)

S2 Fig When the transgene is homozygous, chromatin barrier activity of *Fab-8* is much stronger than when a single copy of the transgene is present. **(A)** Western blot was performed with adult flies. Note that we do detect the expression of DsRed protein in *w*^*1118*^ *Drosophila* using RFP antibody. RT-qPCR was done with adult flies and the relative expression was normalized to Tubulin **(B)** or Rpl32 **(C)**. **(D)** RNA *in situ* hybridization was performed with 12–24 h transgenic embryos.
(TIF)

S3 Fig The ChIP-qPCR expreiments with antibody against RNA Pol II were performed with chromatin isolated from 20–24 h transgenic embryos from heterozygous and homozygous flies. Act5C-TSS region, and NC from a gene desert on *Drosophila melanogaster* chromosome 2R were used as a positive control and a negative control, respectively. Background immunoprecipitation was subtracted from normalized specific ChIP signals at each position.
(TIF)

S4 Fig Determination of transgene copy number by real-time quantitative PCR. Genomic DNAs (gDNAs) were extracted from transgenic adult flies. Quantitative PCR was done with the indicated gDNAs and the primer Fab8-C **(A)**, DsRed2 **(B)** or Ubx-C **(C)**. Transgene copy number is relative to the number of *RpS3* gene copies in each sample. The data are expressed as the Mean ± SD using more than three biological replicates.
(TIF)

S5 Fig Sequences of Hsc70-4 promoter and *F8*^*680*^. The transcription start site of Hsc70-4 promoter is highlighted with red. *Drosophila* CTCF binding sites of *F8*^*680*^ sequence are highlighted with yellow.
(PDF)

S1 Table Primer sequences for RT-qPCR. (DOC)

S2 Table Primer sequences for ChIP-qPCR. (DOC)

S1 Method Biotin-DNA pulldown assay. (DOC)

S2 Method Determination of transgene copy number by real time quantitative PCR. (DOC)
