# Supplementary figures and images for: The Ubx Polycomb response element bypasses an unpaired Fab-8 insulator via cis transvection in Drosophila
Source: PLoS One. 2018 Jun 21;13(6):e0199353. doi: 10.1371/journal.pone.0199353 (PMC6013190; doi:10.1371/journal.pone.0199353)

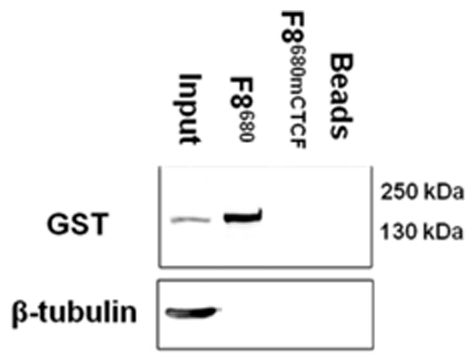

Supplement: S1 Fig — Biotin-DNA pulldown assay indicated that the F8680mCTCF lacking the two CTCF binding sites does not bind to GST-CTCF. Experimental setting for Biotin-DNA pulldown assay is described in S1 Method. (TIF) [file pone.0199353.s001.tif]

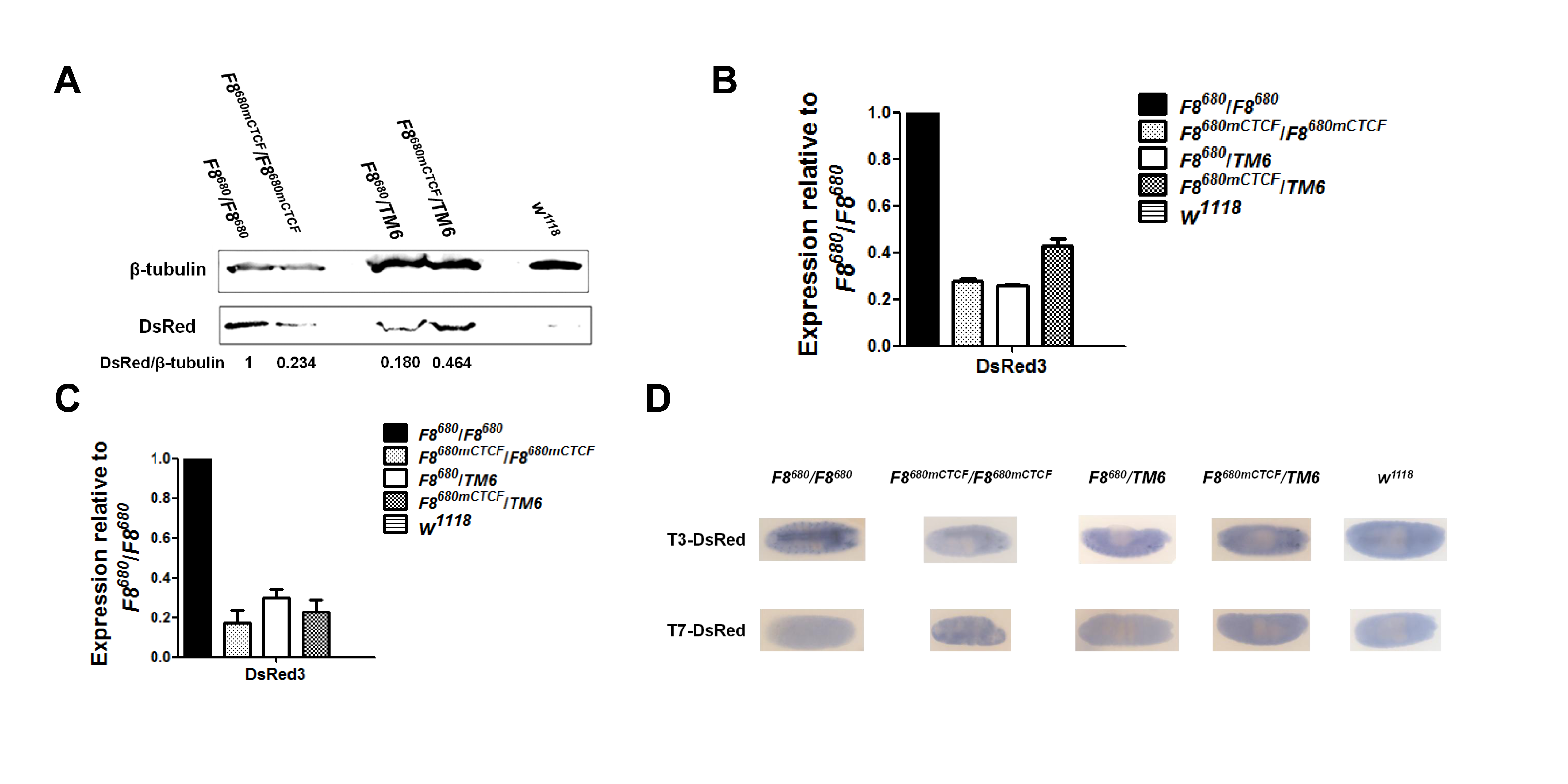

Supplement: S2 Fig — (A) Western blot was performed with adult flies. Note that we do detect the expression of DsRed protein in w1118 Drosophila using RFP antibody. RT-qPCR was done with adult flies and the relative expression was normalized to Tubulin (B) or Rpl32 (C). (D) RNA in situ hybridization was performed with 12–24 h transgenic embryos. (TIF) [file pone.0199353.s002.tif]

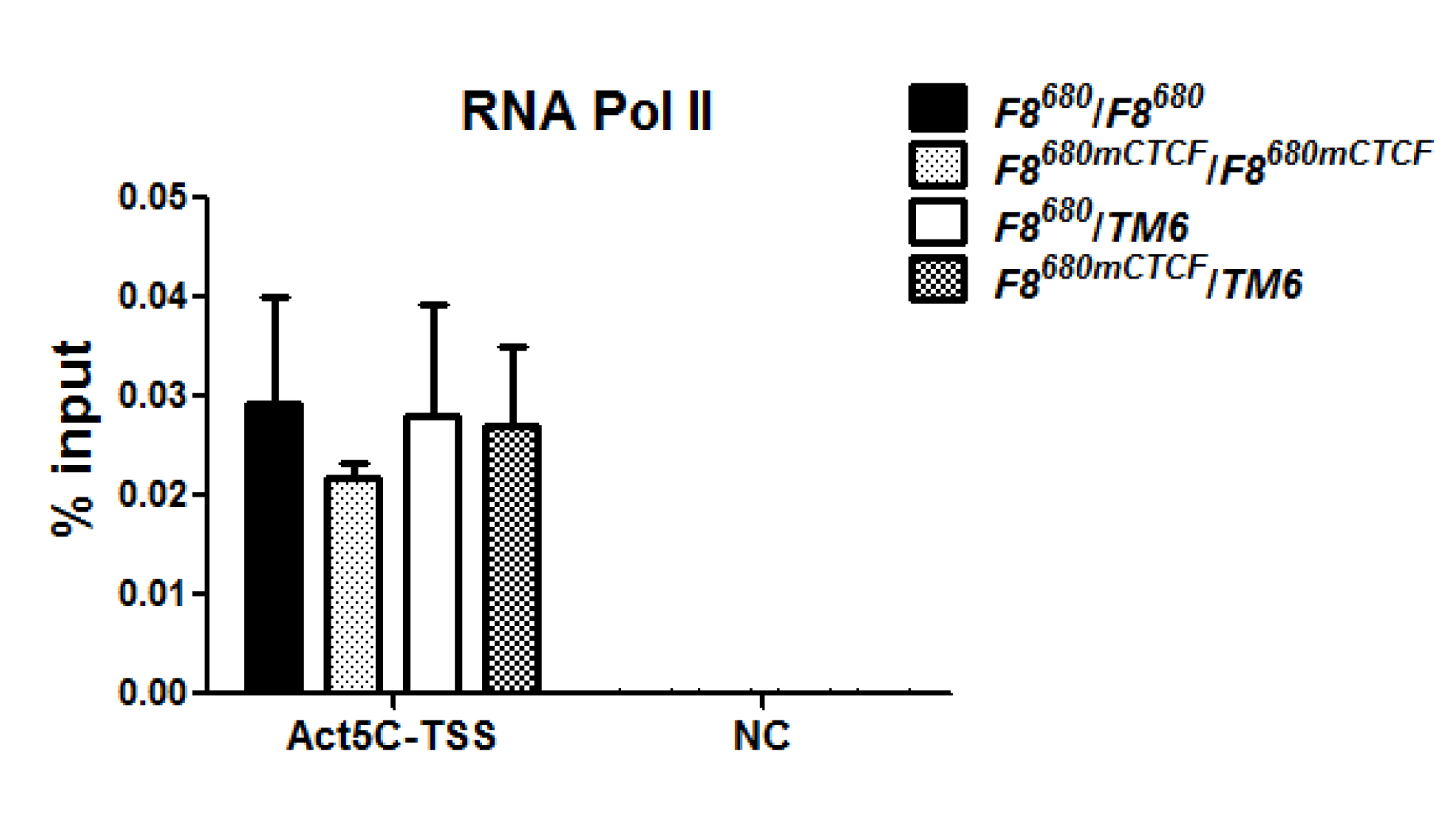

Supplement: S3 Fig — Act5C-TSS region, and NC from a gene desert on Drosophila melanogaster chromosome 2R were used as a positive control and a negative control, respectively. Background immunoprecipitation was subtracted from normalized specific ChIP signals at each position. (TIF) [file pone.0199353.s003.tif]

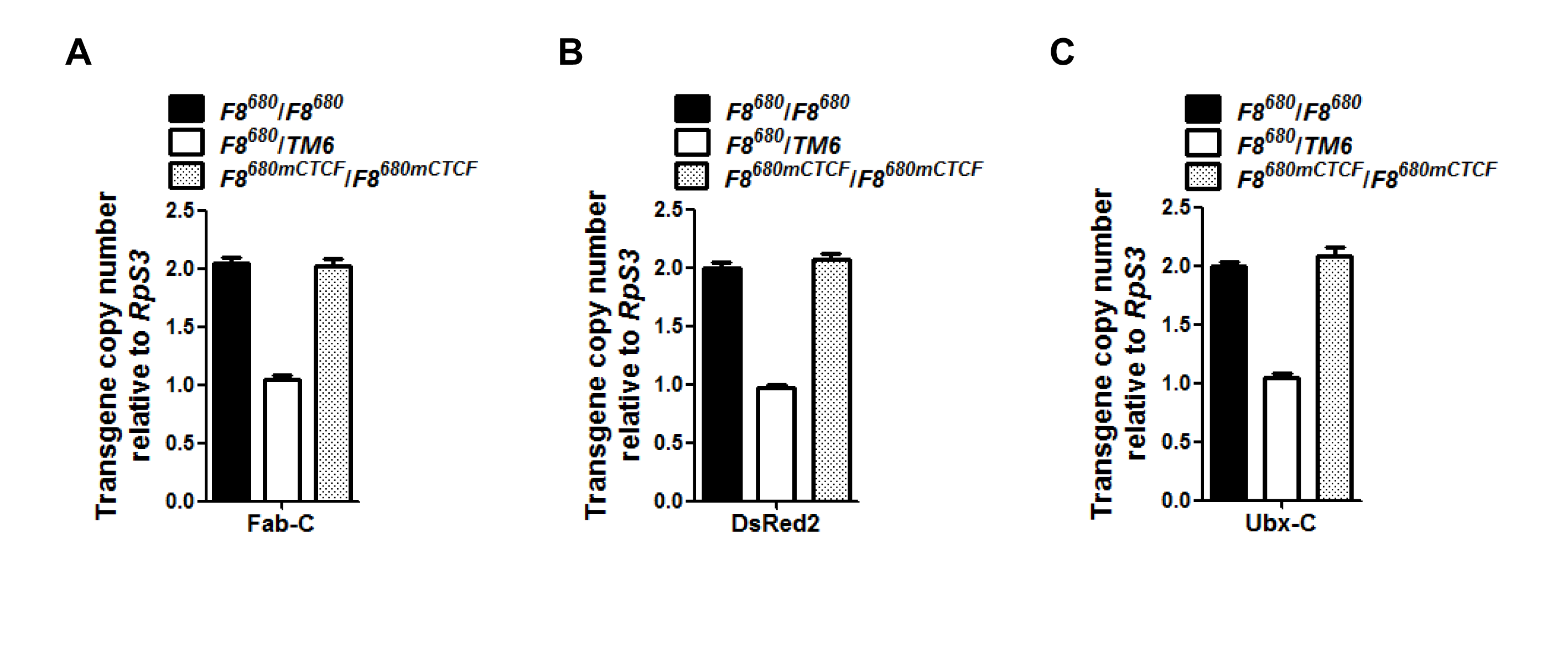

Supplement: S4 Fig — Genomic DNAs (gDNAs) were extracted from transgenic adult flies. Quantitative PCR was done with the indicated gDNAs and the primer Fab8-C (A), DsRed2 (B) or Ubx-C (C). Transgene copy number is relative to the number of RpS3 gene copies in each sample. The data are expressed as the Mean ± SD using more than three biological replicates. (TIF) [file pone.0199353.s004.tif]
