# Supplementary material for: The Ubx Polycomb response element bypasses an unpaired Fab-8 insulator via cis transvection in Drosophila
Source: PLoS One. 2018 Jun 21;13(6):e0199353. doi: 10.1371/journal.pone.0199353 (PMC6013190; doi:10.1371/journal.pone.0199353)
Supplement: S5 Fig — The transcription start site of Hsc70-4 promoter is highlighted with red. Drosophila CTCF binding sites of F8680 sequence are highlighted with yellow. (PDF) [file pone.0199353.s005.pdf]

S5 Fig. Sequences of Hsc70-4 promoter and *F8*^{680}. The transcription start site of Hsc70-4 promoter is highlighted with red. *Drosophila* CTCF binding sites of *F8*^{680} sequence are highlighted with yellow.

365 bp Hsc70-4 promoter

gctcgctgaaaaaggcgaaataatatggcgttttcactaggaaaccgaaatgtgttctactttttccttggttgtcatgggaaagtattcagca
acccccaagtacacaagcaaaatgaaacattcaatatttaatatgtttcaaaggttttctatattttatatttctatacacttaccatctcagcaa
acggttaattttccatctacacgaataacacaacATTTGTTCCATTTTCTCAGTATTACTTCTCCTCTGGCAATCTTTCTATC
GTTTTGGGCACAGTTTTTATATTTTTTGTATATATGTACATCGAGAAAAGGAAAATTAGAATTGTAAAACACAC
CGTTGTAATTCTTTCCAG

F8^{680}

CGTC**AACGCCAACCAGCACAAACACATTCGAATAAGACTCACTCACACAGTGGC
AAGCTGTGCAAGGCATTTGTGTTGGTGAGCAAGCGAAGAGTTCCATTCTCTGCT
TCGAAGTACGAAGAGAGAAAGAGTACTTTAAATTTCCACATTCCCGCCTTGCAG
CGCCACCTGGCCTTGGTAATGTAGAACTAGGAAGGAAAGCACCAACACAAGATG
TCGCTCTCCGACAGTGGACATGTCGCGTAAAAAATGTTCGATAACTTTCAATGGT
TCGATTGAACAGACAATAAGTGTATTTAAGACACCAGTTCTTATATTCAAAAATC
CTAACAACTCACATT**TAAATAAAGGTTAAGATATAATTACATTTCCAACATTAACTT
GATTGTGCCATGTCATTATCTCTTTTCAAAATCTTTCCTTATATTATTGAAACGGATGA
ACGGAAGATGTCTGCATATATAAAAAATGTTTTCGTTAATATTATTTCGTTTGATATA
ATTTTTCATAAATTATAAGACATACATATATGTACATGAAGCATATGTAGACGTACAT
TTTTATAACAATAACTTTCATTATAAATTTGAATTCTGCTTTGATAACAAGAGTACAA
GTTCTGCAAGTAGAAGTTTATGGTATTTGAGAACAATTTTTAGTTTTAAAATAATGAA
CCCAGG

Designations: Transcription Start Site; CTCF binding sites.
