# Supplementary material for: The Ubx Polycomb response element bypasses an unpaired Fab-8 insulator via cis transvection in Drosophila
Source: PLoS One. 2018 Jun 21;13(6):e0199353. doi: 10.1371/journal.pone.0199353 (PMC6013190; doi:10.1371/journal.pone.0199353)
Supplement: S1 Table — (DOC) [file pone.0199353.s006.doc]

**S1 Table. Primer sequences for RT-qPCR**

| Primers | Sequences | References |
| --- | --- | --- |
| Tubulin | F-GCTTTCCCAAGAAGCTCATACA  R-GGTTCAGTGCGGTATTATCCAG | [21] |
| Rpl32 | F-GTTCGATCCGTAACCGATGT  R-CCAGTCGGATCGATATGCTAA | [45] |
| DsRed3 | F-TGTATCCTCGTGATGGCGTG  R-AGTCTTCGTTGTGGCTCGTT |  |

21. Maksimenko O, Kyrchanova O, Bonchuk A, Stakhov V, Parshikov A, Georgiev P. Highly conserved ENY2/Sus1 protein binds to *Drosophila* CTCF and is required for barrier activity. Epigenet. 2014; 9: 1261-1270.

45. Maksimenko O, Bartkuhn M, Stakhov V, Herold M, Zolotarev N, Jox T, et al. Two new insulator proteins, Pita and ZIPIC, target CP190 to chromatin. Genome Res. 2015; 25: 89-99.
