# Supplementary material for: The Ubx Polycomb response element bypasses an unpaired Fab-8 insulator via cis transvection in Drosophila
Source: PLoS One. 2018 Jun 21;13(6):e0199353. doi: 10.1371/journal.pone.0199353 (PMC6013190; doi:10.1371/journal.pone.0199353)
Supplement: S2 Table — (DOC) [file pone.0199353.s007.doc]

**S2 Table. Primer sequences for ChIP-qPCR**

| Primers | Sequences | References |
| --- | --- | --- |
| Ubx-C | F-GTAAAGCGAGAGCGATCCGA  R-TGTTCACGTCGCAAGATCCA |  |
| PRE-RT | F-GCCCTCTCTCTTTTTGAGTTATCG  R-GCCACGCCCCCTTCAC | [21] |
| Rpl32 | F-GTTCGATCCGTAACCGATGT  R-CCAGTCGGATCGATATGCTAA | [45] |
| C1 | F-GGCCGCTCTAGTGGATCTTG  R-GAGCGCTTCCGAAAATGCAA |  |
| Fab8-C | F-ACCGGACTGTAACTAGTCGTC  R-AACTCTTCGCTTGCTCACCA |  |
| C2 | F-TCGCGTTGCATTTTCGGAAG  R-TAGCTCGTTACAGTCCGGTG |  |
| Fab-7 | F-TAAGCCAACTGGTTTCCAACTCT  R-TTGCCCAGGGTAAGTAACGGTAT | [45] |
| CG1354 | F-TTATGCCCGCACAACAATTA  R-GAAATGGGCGTCGTAGCTTA | [21] |
| Hsc-C | F-CTATCGTTTTGGGCGCAGTT  R-CGTCGACATAACTTCGTATAGCA |  |
| 3xP3-C | F-ATAGAGGCGCTTCGTCTACGG  R-GCCGCCCGATTGTTTAGC |  |
| Act5C-TSS | F-AGCGATTGAAAAACGCAGTTA  R-GCACGGTTTGAAAGGAATGA | [37] |
| F10 | F-AATTGGTTTCCAGGGATCTGC  R-ATCCAAAGGAGGCAAAGGAAC | [46] |
| F19 | F-ACACTGCGAGCGCCTCACACGC  R-CCTAGGTGAATGTGCGGCACAC | [46] |
| F22 | F-CAGTTGATGGGATGAATTTGG  R-TGCCTGTGGTTCTATCCAAAC | [46] |
| DsRed2 | F-ACCTTTGCCATTTGCTTGGG  R-CGCCACCGTCTTCAAAGTTC |  |
| DsRed3 | F-TGTATCCTCGTGATGGCGTG  R-AGTCTTCGTTGTGGCTCGTT |  |
| NC | F-AAACGCCCATAACTCGGTAAAT  R-AGACTGTTAATGATGTTGCTGTTG |  |
| RpS3 | F-TCTTTCTTTTCTGCGCACCA  R-TCGCATTCATTTTGACGTCG | [47] |

21. Maksimenko O, Kyrchanova O, Bonchuk A, Stakhov V, Parshikov A, Georgiev P. Highly conserved ENY2/Sus1 protein binds to *Drosophila* CTCF and is required for barrier activity. Epigenet. 2014; 9: 1261-1270.

37. Gaertner B, Johnston J, Chen K, Wallaschek N, Paulson A, Garruss AS, et al. Poised RNA polymerase II changes over developmental time and prepares genes for future expression. Cell Rep. 2012; 2: 1670-1683.

45. Maksimenko O, Bartkuhn M, Stakhov V, Herold M, Zolotarev N, Jox T, et al. Two new insulator proteins, Pita and ZIPIC, target CP190 to chromatin. Genome Res. 2015; 25: 89-99.

46. Papp B, Muller J. Histone trimethylation and the maintenance of transcriptional ON and OFF states by TrxG and PcG proteins. Gene. Dev. 2006; 20: 2041-2054.

47. Bel Y, Ferre J, Escriche B. Quantitative real-time PCR with SYBR Green detection to assess gene duplication in insects: Study of gene dosage in *Drosophila melanogaster* (Diptera) and in *Ostrinia nubilalis* (Lepidoptera). BMC Res. Notes. 2011; 4: 84-91.
