# Supplementary material for: The Ubx Polycomb response element bypasses an unpaired Fab-8 insulator via cis transvection in Drosophila
Source: PLoS One. 2018 Jun 21;13(6):e0199353. doi: 10.1371/journal.pone.0199353 (PMC6013190; doi:10.1371/journal.pone.0199353)
Supplement: S1 Method — (DOC) [file pone.0199353.s008.doc]

**S1 Method. Biotin-DNA pulldown assay**

The cDNA of full-length *Drosophila* CTCF with N-terminal GST tag was synthesized using the primers (5’ATGCCAAGGAGGACAAAAAAGGACGAGGA3’ and 5’CTAAGAGTCCTGCTCAATCATATCCATCAGC3’) and sub-cloned into pCDH vector (CD513B-1, SBI) with ClonExpress II One Step Cloning Kit (Vazyme) and a Kozak sequence (GCCACC) was added to immediately upstream of the start codon. Biotin-labeled *F8^680^* and *F8^680mCTCF^* probes were PCR-synthesized using primers (5’CGTCAACGCCAACCAGCAC3’ and 5’CCTGGGTTCATTATTTTAAAAC3’) biotinylated at their 5’ ends and diluted in 10 mM Tris-HCl, 10 mM MgCl_2_, 25 mM NaCl. HEK293T cells transfected with pCDH-GST-CTCF were lysed in lysis buffer containing 50 mM Tris-HCl, pH 7.4, 150 mM NaCl, 5 mM MgCl_2_, 10% glycerol, 1% NP-40 and 1× protease inhibitor cocktail for 30 min on ice. Cell lysate was incubated with 2 μg biotin-labeled probes overnight at 4°C. The high capacity streptavidin agarose resin (Pierce) was added to the mixture and incubated at 4°C for another 4 h. the resin was subsequently precipitated by centrifuging at 10,000 g for 30 s and washed four times in lysis buffer containing 500 mM NaCl at 4°C. Then, the samples were directly boiled in 5×SDS loading buffer and subjected to SDS-PAGE followed by immunoblot analysis using GST antibody (Transgen, China).
