# Supplementary material for: The Ubx Polycomb response element bypasses an unpaired Fab-8 insulator via cis transvection in Drosophila
Source: PLoS One. 2018 Jun 21;13(6):e0199353. doi: 10.1371/journal.pone.0199353 (PMC6013190; doi:10.1371/journal.pone.0199353)
Supplement: S2 Method — (DOC) [file pone.0199353.s009.doc]

**S2 Method. Determination of transgene copy number by real-time quantitative PCR.**

Genomic DNA (gDNA) was isolated from heterozygous and homozygous transgenic adult flies using the DNeasy Tissue Kit (Qiagen, Germany) according to the manufacturer’s instructions. A twofold dilution series of genomic DNA was used as the template. The real-time quantitative PCR analyses were performed using a Roche LightCycler 480 equipment. The PCR efficiency for the transgene and the copy number control gene were calculated, and then the ratio of the copy number of the transgene to the reference gene was calculated. The autosomal single copy gene *RpS3* was used as a reference genes. Primers sequences are given in S2 Tables.
